# Intestinal Ischemia/Reperfusion Injury: Mechanisms, Diagnosis, and Therapeutic Advances

**DOI:** 10.7150/ijbs.130025

**Published:** 2026-03-30

**Authors:** Fan Deng, Qi-Shun Sun, Sun Xie, Ling Wu, Shi-Ying He, Bo-Wei Zhou, Zhen Hu, Wan-Yi Lian, Bing-Jie Li, Cai Li, Wei -Feng Liu, Jing-Juan Hu, Ke-Xuan Liu

**Affiliations:** Department of Anesthesiology, Nanfang Hospital, Southern Medical University, Guangzhou, Guangdong, China.

**Keywords:** intestinal ischemia-reperfusion, extraintestinal organs injury, diagnostic strategies, biomarkers, therapeutic approaches

## Abstract

Intestinal ischemia/reperfusion (I/R) injury is a critical clinical syndrome precipitated by the restoration of blood flow following intestinal ischemia, a common occurrence in perioperative settings such as abdominal aortic surgery, hemorrhagic shock, and cardiopulmonary bypass. This injury extends beyond the local gut, as the disruption of the intestinal mucosal barrier facilitates bacterial and endotoxin translocation into the systemic circulation, triggering a systemic inflammatory response that can progress to sepsis and multiple organ dysfunction syndrome (MODS). Despite advances in critical care, the mortality rate associated with severe intestinal I/R injury remains formidable. The microcirculatory disturbances and organ damage following intestinal I/R involve complex pathological processes, including metabolic injury and oxidative stress. In recent years, rapid developments in the understanding of cell death mechanisms, gut microbiota, microRNAs, and fundamental medical technologies have significantly advanced research on the prevention and treatment of I/R injury. This review aims to comprehensively summarize the occurrence and progression of intestinal I/R, its impact on extraintestinal organ injury, diagnostic strategies and biomarkers, as well as current treatment methods, thereby providing guidance for the future prediction, diagnosis, and treatment of intestinal I/R injury.

## Introduction

Intestinal ischemia/reperfusion (I/R) injury frequently arises in perioperative settings, particularly following high-risk procedures such as severe trauma, hemorrhagic shock, severe infection, intestinal obstruction, small bowel transplantation, abdominal aortic surgery, and cardiopulmonary bypass. In such clinical scenarios, intestinal I/R injury substantially contributes to patient morbidity and mortality^1^. Despite progress in modern medical technology, the in-hospital mortality rate associated with I/R injury remains alarmingly high, ranging from 60% to 80%^2^, ^3^, and its incidence continues to increase^3^, ^4^. Emerging evidence suggests that susceptibility to intestinal I/R injury may be influenced by biological sex, with females exhibiting a relatively greater tolerance than males^5^. At the core of intestinal I/R pathophysiology lies profound microcirculatory dysfunction, characterized by impaired nutrient perfusion, endothelial and epithelial injury, inflammatory cell activation, and loss of epithelial barrier integrity^6^. Importantly, microcirculatory failure represents the initiating event that converts a localized ischemic insult into a systemic inflammatory disorder. During ischemia, metabolic derangements and the accumulation of toxic metabolites prime the tissue for injury. Upon reperfusion, the abrupt restoration of blood flow triggers excessive production of reactive oxygen species (ROS) and inflammatory mediators, leading to epithelial cell death and breakdown of the intestinal barrier. This disruption facilitates the translocation of gut-derived bacteria, endotoxins, and damage-associated molecular patterns (DAMPs) into the systemic circulation and mesenteric lymph. Through this gut-centered inflammatory amplification axis, intestinal I/R injury propagates systemic immune activation, distant organ inflammation, and ultimately multiple organ dysfunction or failure^7^-^9^. Thus, intestinal I/R injury serves as a critical mechanistic link between localized ischemic stress and systemic inflammatory syndromes, highlighting the intestine as a central driver rather than a passive victim of multi-organ injury.

In recent years, rapid advances in the understanding of cell death mechanisms, gut microbiota, microRNA biology, and related biomedical technologies have spurred numerous innovative studies aimed at preventing or mitigating I/R injury, many of which have been published in high-impact journals. This review aims to comprehensively summarize current knowledge on the pathogenesis and progression of intestinal I/R injury, its systemic effects on distant organs, available diagnostic strategies and biomarkers, as well as existing and emerging therapeutic approaches. Ultimately, this synthesis aims to provide a foundation for improving the prediction, diagnosis, and clinical management of intestinal I/R injury.

## Pathophysiology of intestinal I/R-induced intestinal injury

**Figure 1:**
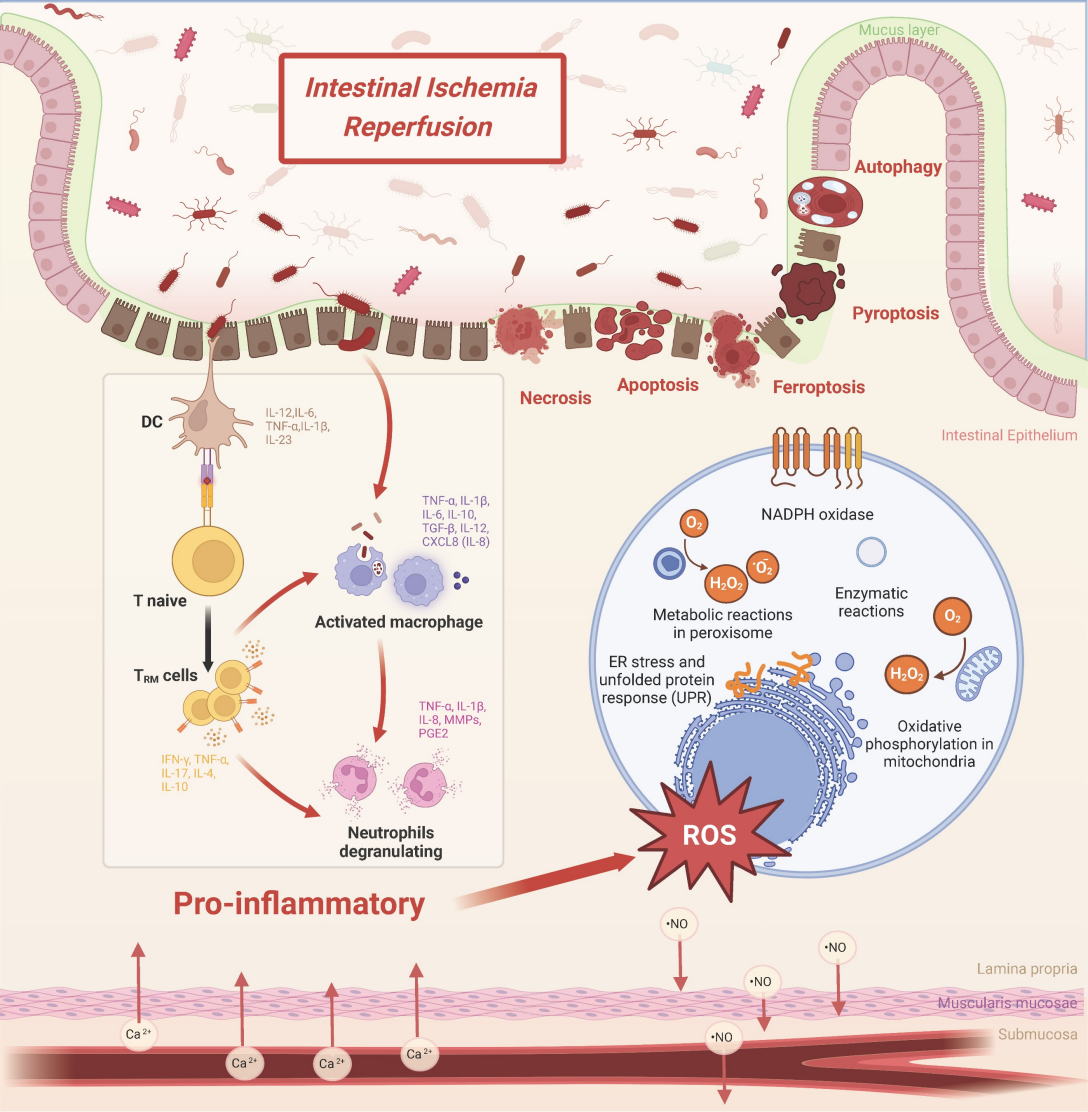
** Pathophysiological mechanisms of intestinal ischemia-reperfusion (I/R) injury.** Hypoxia-reoxygenation cycles drive oxidative stress through NADPH oxidase activation, mitochondrial dysfunction, and endoplasmic reticulum (ER) stress. Immune activation involves coordinated infiltration of macrophages (releasing IL-6, TNF-α, and matrix metalloproteinases), neutrophils (producing reactive oxygen species and nitric oxide), and tissue-resident memory T cells (TRM; IL-17/IFN-γ), perpetuating mucosal inflammation. Barrier failure mechanisms arise from convergent cell death pathways: necrosis (with release of HMGB1 and ATP), NOX2-mediated apoptosis, NLRP3/gasdermin D-dependent pyroptosis, and ferroptosis driven by glutathione peroxidase 4 deficiency. Structural deterioration is characterized by depletion of goblet cells and disruption of the mucus layer.

The core pathophysiology of intestinal I/R injury lies in the sequential collapse of multi-layered defense barriers. This directly leads to uncontrolled systemic inflammation. This entire process is not a singular event but rather a stereoscopic, network-like cascade initiated by ischemia and amplified upon reperfusion (Figure 1). The primary pathological manifestation is the comprehensive disruption of intestinal barrier function, encompassing the mechanical, chemical, microbial, and immune components. This breach facilitates the translocation of luminal bacteria and endotoxins, thereby driving systemic inflammatory response syndrome (SIRS) and multiple organ dysfunction^10^-^12^. Therefore, deciphering this intricate network is essential for developing effective therapeutic strategies.

## Breach of the intestinal barrier: from structural destruction to ecological collapse

The integrity of the intestinal barrier serves as the primary line of defense in maintaining internal homeostasis, which is subjected to multi-dimensional assault during I/R injury.

### Direct destruction of the mechanical and chemical Barriers

Intestinal epithelial cells, the core of the physical barrier, are primary targets of I/R injury. Ischemia directly induces a cellular energy crisis and acidosis, initiating the injury program. The subsequent burst of ROS upon reperfusion delivers the decisive blow^13^-^15^. This not only directly damages cellular structures but, more critically, triggers various regulated cell death pathways. These pathways include apoptosis, necroptosis, pyroptosis, ferroptosis, and autophagy^16^-^23^. Ultimately, this leads to extensive epithelial sloughing. Concurrently, the secretion of the protective mucus layer is reduced, and the production of chemical barrier components such as antimicrobial peptides and digestive enzymes is impaired^24^-^26^, rendering the exposed mucosa more vulnerable to attack.

### Dysbiosis of the microbial barrier: from symbiotic partner to injury accomplice

A healthy gut microbiota constitutes a vital biological barrier. The hypoxic and inflammatory milieu created by I/R rapidly induces gut dysbiosis. Its characteristic is a decrease in beneficial bacteria and an overgrowth of potentially pathogenic ones^27^, ^28^. This shift is not merely a passive consequence but actively amplifies injury as an amplifier. For instance, succinate produced by dysbiotic microbiota can exacerbate remote lung injury^29^, while specific probiotic strains (e.g., *L. murinus*) or their beneficial metabolites (e.g., capsiate, CAT) can exert protective effects by inducing IL-10 secretion^30^ or upregulating glutathione peroxidase 4 (GPX4) to inhibit ferroptosis^21^. Furthermore, gut microbiota-derived arginine metabolism has also been shown to mitigate intestinal I/R injury^31^. This highlights the gut microbiota as a crucial and modulable target.

### Hyperactivation of the immune barrier: from defensive guard to inflammatory storm

The intestinal immune system is dramatically activated during I/R. DAMPs released from injured epithelium and invading microbial products, through pattern recognition receptors such as Toll-like receptors (TLRs)—particularly the TLR4/MyD88 axis—ignite the innate immune response^32^-^34^. This leads to massive infiltration of neutrophils and macrophages, which release potent weapons including neutrophil extracellular traps (NETs), causing severe “collateral damage” while attempting to clear threats^35^-^39^. Notably, certain immune cell subsets (e.g., ILC3s) can also secrete cytokines like IL-22 to attempt repair, underscoring the complexity of the immune response^40^, ^41^.

## Core signaling pathways: regulatory hubs of the injury network

The aforementioned barrier disruption processes are precisely orchestrated by several highly conserved core signaling pathways, which constitute promising molecular targets for intervention. Among them, the pro-inflammatory MAPK and NF-κB pathways, together with the antioxidant nuclear factor erythroid 2-related factor 2 (Nrf2) pathway, form a central regulatory network that determines cellular fate.

### Key signaling pathways governing inflammation, death, and defense

The p38 MAPK/JNK pathways play a central role in stress response and cell death, and their inhibitors can significantly attenuate epithelial apoptosis^42^, ^43^. STING deficiency has been shown to alleviate I/R injury via JAK2/STAT3-mediated macrophage polarization and autophagy^44^. Studies indicate that the p38 MAPK inhibitor SB239063 mitigates lung injury by decreasing AQP4 levels^45^. Anesthetic agents such as propofol and dexmedetomidine also protect against I/R injury by inhibiting mitochondrial apoptosis and inflammation mediated by this pathway^46^, ^47^. Recent research further demonstrates that dexmedetomidine-loaded polypeptide nanospheres (PNM@Dex) alleviate intestinal I/R injury by modulating autophagy and inflammation^48^. In parallel, NF-κB acts as the master switch for pro-inflammatory gene expression, and its hyperactivation drives the production of inflammatory mediators such as TNF-α and IL-1β^49^-^53^. These two pathways often form positive feedback loops, mutually exacerbating the injury. Counteracting the pro-inflammatory pathways, the Nrf2 pathway serves as the central hub for cellular antioxidant defense^54^. Its activation upregulates a suite of cytoprotective proteins to combat ROS insult. For example, isoquercitrin mitigates intestinal I/R injury by regulating intestinal flora and activating the Nrf2/HO-1 signaling axis^55^. The significantly aggravated injury observed in Nrf2-knockout animals following I/R confirms its critical protective role^56^.

### TLR signaling: the upstream gatekeeper

The activation of these core pathways is often initiated by upstream pattern recognition receptors, among which the TLR family is particularly critical. TLR4 serves as a primary sensor for endogenous DAMPs and exogenous lipopolysaccharide (LPS). It transduces signals via adaptor proteins such as MyD88, thereby activating downstream NF-κB and MAPK pathways. This process ultimately launches the inflammatory cascade^57^. Modulating TLR4 signaling (e.g., with melatonin or shRNA-mediated silencing^39^) has proven effective in mitigating injury. Intriguingly, TLR2 signaling may, in specific contexts, exert protective effects by inducing anti-inflammatory factors like IL-10^58^, suggesting context-dependent functionalities among TLR family members.

In summary, intestinal I/R injury originates from epithelial metabolic and oxidative crises. This progresses through cell death, microbial dysbiosis, and immune hyperactivation, culminating in the complete loss of the physical and functional integrity of the intestinal barrier. Intestinal barrier dysfunction and immune hyperactivation are not independent processes. Instead, they interact within a self-reinforcing pathological cycle. This cycle is initiated by oxidative stress, further amplified through inflammation driven by the TLR/NF-κB pathway, and exacerbated by the suppression of protective mechanisms such as the Nrf2 pathway. Together, these interactions create a progressively worsening pathological network. Therefore, preserving the intestinal barrier and modulating the core signaling pathways represent the key strategic approaches to interrupt this lethal cascade.

## Intestinal I/R-induced extraintestinal organ injury

**Figure 2:**
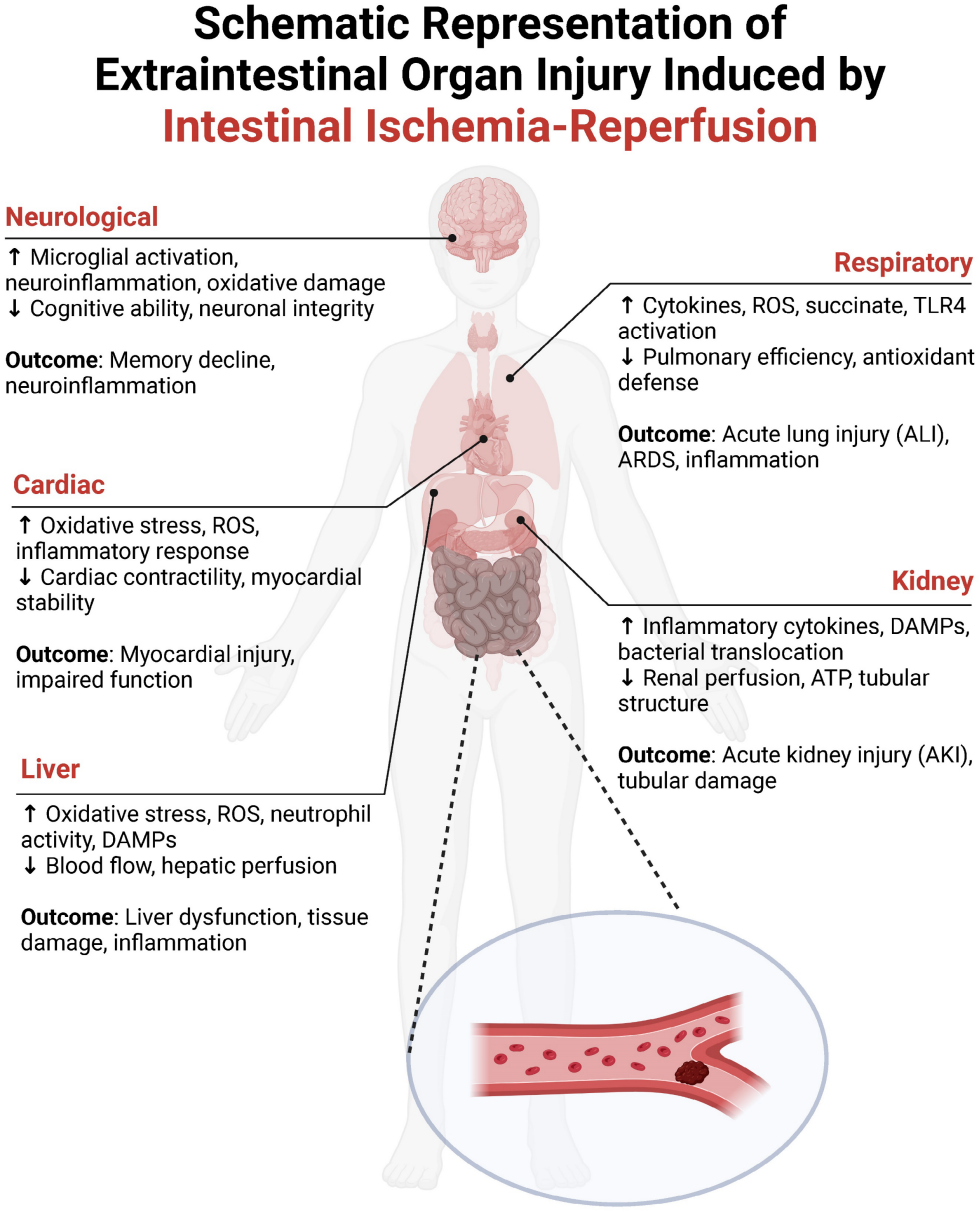
**Schematic representation of multiorgan injury following intestinal ischemia-reperfusion (I/R)**. This figure delineates systemic pathophysiology triggered by intestinal I/R. **Neurological system**: Neuroinflammation (microglial activation, oxidative stress) impairs neuronal integrity and cognitive function. **Cardiac system**: Oxidative stress and reactive oxygen species (ROS)-driven inflammation induce myocardial dysfunction and contractile impairment. **Hepatic system**: ROS-mediated damage, neutrophil activity, and ischemic hypoperfusion drive hepatocellular necrosis and inflammation. **Respiratory system**: Succinate accumulation and TLR4/ROS signaling promote acute lung injury/acute respiratory distress syndrome (ALI/ARDS) through cytokine storm and alveolar damage. **Renal system**: Damage-associated molecular patterns (DAMPs)-mediated inflammation and hypoperfusion trigger acute kidney injury (AKI) with characteristic tubular necrosis.

Figure 2 illustrates the systemic communication between the intestine and distant organs during I/R injury, outlining the key pathophysiological mechanisms and their consequences. The ischemic gut generates a substantial amount of ROS, which, along with a surge of inflammatory mediators (e.g., TNF-α, IL-6), enters the circulation. This “toxic effluent” can damage pulmonary vasculature and alveoli (leading to acute lung injury), constrict renal vessels and harm tubular epithelium (causing acute kidney injury), alter myocardial electrophysiology and contractility (inducing cardiac dysfunction), promote hepatocyte death (disrupting liver metabolism), and compromise the blood-brain barrier (resulting in cerebral edema and neuronal damage). Thus, intestinal I/R is a potent driver of multi-organ dysfunction, underscoring the need for a mechanistic understanding to guide clinical intervention.

## Intestinal I/R and liver injury

The intimate anatomical and functional link between the intestine and liver via the portal system makes the liver a primary target for gut-derived insults. Intestinal I/R can reduce hepatic perfusion by up to 80%^59^, contributing directly to acute hepatic dysfunction^60^. More critically, the breach of the intestinal barrier allows microbial products (e.g., LPS) and DAMPs (e.g., HMGB1, histones) to translocate via the portal vein to the liver^61^-^64^. This influx activates hepatic innate immunity, notably via TLR4, leading to neutrophil recruitment. Neutrophils exacerbate injury through ROS release, protease secretion, and NETosis^63^, ^65^. The resulting sterile inflammation, combined with direct oxidative stress from systemic ROS^66^, ^67^, drives hepatocyte apoptosis and necrosis, compromising the liver's vital metabolic and detoxifying functions.

## Intestinal I/R and kidney injury

Intestinal I/R precipitates acute kidney injury (AKI) through combined hemodynamic and inflammatory mechanisms. There is a significant reduction in renal blood flow and tissue ATP levels following intestinal insult, impairing tubular function^68^. Histologically, the kidney displays changes akin to sepsis-induced AKI, including tubular dilation, epithelial flattening, and cast formation^69^. The gut-kidney axis plays a central role: intestinal barrier failure leads to the translocation of bacteria, toxins, and DAMPs, which directly damage renal tissue and incite local inflammation. Conversely, AKI-induced uremia and systemic inflammation further worsen intestinal integrity, creating a vicious cycle that fuels progressive renal dysfunction^70^. Currently, research predominantly focuses on the acute manifestations of AKI triggered by I/R injury, while its chronic progression remains poorly understood. This underscores the urgent need for further investigation into the long-term effects of intestinal I/R on renal health to develop effective preventive and management strategies.

## Intestinal I/R and heart injury

Myocardial injury following intestinal I/R manifests as structural disarray, including myofiber derangement, interstitial edema, and nuclear pyknosis^71^, ^72^. The primary drivers are systemic inflammation and profound oxidative stress. The influx of inflammatory mediators and ROS during reperfusion disrupts cardiomyocyte electrophysiology and contractility, leading to arrhythmias and depressed function^73^. Oxidative stress also alters the expression of cardiac genes related to antioxidant defense^73^. Therapeutic strategies that enhance antioxidant capacity (e.g., hyperbaric oxygen^74^), modulate metabolism and inflammation (e.g., adenosine^75^), or reduce cardiac workload (e.g., atenolol^72^) have shown protective potential, highlighting the importance of targeting metabolic instability and oxidative damage.

## Intestinal I/R and lung injury

The lung, with its extensive capillary network, is highly susceptible to I/R-induced damage, making acute lung injury/ARDS a common severe complication. The gut-lung axis is pivotal: microbial metabolites like succinate, whose balance is disrupted during I/R, can travel to the lungs, polarize alveolar macrophages via SUCNR1, and exacerbate injury^29^, ^76^. Moreover, bacterial translocation and systemic DAMPs (e.g., cold-inducible RNA-binding protein (CIRP) activate pulmonary TLR4, driving NF-κB and p38 MAPK pathways that intensify inflammation and endothelial damage^34^, ^77^, ^78^.

Various interventions targeting these inflammatory pathways show therapeutic potential. The p38 MAPK inhibitor SB239063 alleviates I/R-induced lung injury by reducing IL-1β expression^43^. Similarly, curcumin and the sirtuin inhibitor FK866 attenuate lung inflammation and apoptosis via fine-tuning NF-κB signaling and reducing pro-inflammatory cytokine release^50^, ^53^. Beyond direct pathway inhibition, regulating related cellular functions is another important approach. For instance, I/R-associated oxidative stress is closely connected to mast cell degranulation. Agents such as propofol, sevoflurane, and resveratrol exert protective effects by stabilizing mast cells^79^-^81^. Ischemic preconditioning (IPO) and immediate ischemic postconditioning (IPC) during reperfusion also mitigate lung injury by suppressing peroxide generation, restoring superoxide dismutase (SOD) activity, and inhibiting inflammatory^82^.

In addition to inflammation, oxidative stress and its mediated forms of cell death are key mechanisms of lung injury. Natural extracts, including Ginkgo biloba extract (EGb 761), artemisinin, and cilostazol, also exhibit lung-protective effects through antioxidant mechanisms^83^-^85^. Recently, the role of ferroptosis in ALI has garnered significant attention. Nrf2 can inhibit ferroptosis by upregulating the expression of molecules such as TERT and SLC7A11, thereby reducing alveolar epithelial cell apoptosis^86^. Furthermore, studies have revealed that the YAP/Nrf2 pathway alleviates acute lung injury by suppressing ferroptosis^87^. Additionally, celastrol has been shown to attenuate ferroptosis-mediated intestinal I/R-induced acute lung injury via Hippo-YAP signaling^88^. Inhibitor of apoptosis-stimulating protein of p53 and isoliquiritin also suppress ferroptosis by attenuating aberrant Fe^2+^ accumulation in lung tissue^89^, ^90^. Moreover, the PKCβII/p66Shc pathway is involved in regulating apoptosis, and its inhibition reduces caspase-3 cleavage^78^, ^91^. Notably, the endogenous antimicrobial peptide Beta-defensin-2 is upregulated following I/R injury, and moderate suppression of this upregulation may attenuate lung damage by inhibiting excessive inflammation and oxidative stress^92^, ^93^, highlighting its complex role in injury pathogenesis. Recent studies have further expanded the intervention approaches. For instance, electroacupuncture has been shown to alleviate acute lung injury through the vagus-sympathetic nerve pathway^94^, providing new evidence for lung protection strategies targeting neuroimmune regulation.

Collectively, lung injury following I/R involves the interplay of gut-lung axis dysfunction, activation of inflammatory signaling, oxidative stress, and multiple cell death pathways. Current research not only deepens the understanding of injury mechanisms but also provides a scientific basis for developing combined therapeutic strategies targeting inflammatory pathways, antioxidant defense, and the regulation of programmed cell death.

## Intestinal I/R and brain injury

Intestinal I/R can induce remote brain injury characterized by neuroinflammation, blood-brain barrier disruption, and cognitive impairment. Mechanistically, this process is mediated by circulating factors, with enterogenic exosomes identified as key carriers of injurious signals that activate brain microglia. For instance, a recent study demonstrated that intestinal I/R injury influences hyaluronan homeostasis in the rat brain^95^. Microglial activation peaks around 24 hours post-reperfusion and is a central driver of neuronal oxidative stress and memory deficits^96^. The cerebrovascular barrier is compromised earlier, within hours of reperfusion^97^. Hall *et al*. previously reported that a rat model subjected to one hour of intestinal ischemia followed by two hours of reperfusion did not exhibit significant damage in the central nervous system^98^. This lack of damage may be attributed to the short duration of reperfusion, which likely did not reach the critical threshold necessary for inducing brain injury. Subsequent research has highlighted that enterogenic exosomes serve as key mediators of I/R-induced brain injury, contributing to memory decline through microglial activation^99^. Melatonin and small extracellular vesicles derived from *Akkermansia muciniphila* can attenuate microglial activation by modulating TLR pathways, thereby alleviating neuroinflammation and cognitive impairment^57^, ^100^. Additionally, the locus coeruleus norepinephrine (LCNE) system plays a significant role in mitigating cerebral injury. Dexmedetomidine preconditioning acts by activating central α2-adrenergic receptors, which inhibits the LCNE system, reduces brain norepinephrine levels, and subsequently stimulates cholinergic anti-inflammatory pathways to improve cognitive function^98^.

## The diagnosis and biomarkers of intestinal injury

Acute mesenteric ischemia (AMI), a critical manifestation of intestinal I/R injury, results from the abrupt interruption of mesenteric blood flow^1^. Despite its rarity, AMI carries a mortality rate exceeding 60%, largely attributable to diagnostic delays stemming from its nonspecific and often atypical clinical presentation. The classic symptom of severe, poorly localized abdominal pain is less common than more insidious "chronic-acute" forms^101^, ^102^. Therefore, heightened clinical suspicion, coupled with the judicious use of modern imaging and biomarkers, is paramount for timely diagnosis and improved outcomes.

## Imaging modalities

Imaging is the cornerstone for confirming the diagnosis and assessing the extent of intestinal I/R injury.

Computed Tomography Angiography (CTA): CTA, particularly with multi-detector CT (MDCT) technology, is the first-line imaging modality for suspected AMI. It offers exceptional accuracy (sensitivity > 93%, specificity up to 100%) in detecting vascular occlusion, bowel wall abnormalities (e.g., edema, pneumatosis), and secondary signs of ischemia^103^. Its speed and widespread availability make it indispensable for urgent evaluation.

Magnetic Resonance Angiography (MRA): MRA serves as a valuable alternative, especially for patients with contraindications to iodinated contrast or for evaluating chronic mesenteric ischemia^104^. It provides excellent visualization of the proximal mesenteric vasculature. However, its utility may be limited in assessing distal branch occlusions or non-occlusive ischemia^105^, ^106^. Advanced techniques like 4D flow MRI are enhancing its diagnostic role^104^, ^107^.

Doppler Ultrasound: While not a primary tool for diagnosing acute AMI due to operator dependency and technical limitations in an acute setting^108^, Doppler ultrasound plays a significant role in the initial assessment and screening of chronic mesenteric ischemia. It effectively evaluates stenosis in the superior mesenteric and celiac arteries by measuring blood flow velocities^109^-^111^.

Abdominal Radiography (X-ray): Plain abdominal X-rays have very limited diagnostic value in AMI. Findings are often non-specific and appear only in late stages (e.g., pneumoperitoneum from perforation), making it an unreliable tool for exclusion^108^, ^112^. Its use in this context is generally discouraged. In contrast, abdominal CT imaging is highly effective in detecting critical indicators such as pneumatosis intestinalis and portomesenteric venous gas, rendering it superior for this purpose. Therefore, abdominal CT should be prioritized for suspected AMI to enhance diagnostic accuracy and timeliness, ultimately optimizing patient care and treatment outcomes.

## Circulating biomarkers

Biomarkers offer complementary, non-invasive tools to raise suspicion and support diagnosis, though none are pathognomonic alone.

D-dimer: D-dimer testing plays a crucial role in diagnosing AMI; however, its limitations in specificity and sensitivity necessitate the use of supplementary diagnostic methods^113^, ^114^. While a normal level may help rule out ischemia in low-risk patients, elevated levels (e.g., > 0.9 mg/L or > 1000 ng/ml) significantly increase the probability of AMI, especially when combined with risk factors like atrial fibrillation^115^, ^116^. It is best used as an adjunct to clinical assessment and imaging.

Intestinal fatty acid-binding protein (I-FABP): I-FABP, a cytosolic protein abundant in enterocytes, is released rapidly upon intestinal epithelial injury. It shows high sensitivity for detecting intestinal ischemia, particularly in conditions like strangulating bowel obstruction^117^, ^118^. Its detection in plasma or, notably, in urine provides a promising non-invasive method for early suspicion^117^, ^118^, though standardized assays are needed for widespread clinical adoption.

Lactate: Serum or arterial lactate is a marker of tissue hypoperfusion and anaerobic metabolism. Elevated lactate is a concerning sign in suspected AMI, indicating advanced ischemia and potential bowel necrosis^119^-^122^. Although serum lactate measurements may lack absolute specificity for diagnosing AMI, they remain crucial for assessing the severity of the condition. Specifically, patients exhibiting symptoms of peritoneal inflammation and abdominal infections, along with lactate concentrations exceeding 2 mmol/L—especially when clinical suspicion focuses on peritonitis and small bowel compromise leading to necrosis—are strongly advised to undergo surgical exploration^123^. Nevertheless, it is imperative to exercise caution, as an isolated elevation in lactate levels, without supporting clinical evidence, is insufficient to differentiate between the onset of ischemia and irreparable intestinal damage.

Other Promising Biomarkers: Citrulline: A low plasma citrulline level reflects reduced functional enterocyte mass and shows promise as a marker of intestinal compromise^124^, ^125^. Procalcitonin (PCT): Serum PCT demonstrates predictive value for bowel ischemia and necrosis in cases of bowel obstruction^126^-^128^. Inflammatory Markers: The measurement of plasma IL-6 upon admission may serve as a predictive marker for intestinal ischemia and assist in the acute diagnosis of clinically suspicious cases^129^-^131^. Additionally, immature granulocyte count and delta neutrophil index are reliable indicators for evaluating intestinal necrosis in cases of mesenteric ischemia^132^, ^133^. Tissue-Specific Proteins: SM22 (released from damaged intestinal smooth muscle) and alpha-glutathione S-transferase are being explored as more specific indicators of bowel wall injury^134^, ^135^.

The diagnosis of intestinal I/R injury requires a multimodal approach. A high index of clinical suspicion must trigger timely CTA imaging for definitive anatomical assessment. Biomarkers like D-dimer, I-FABP, and lactate serve as valuable adjuncts to raise early suspicion, triage patients, and gauge disease severity. The future lies in validating panels of biomarkers and integrating them with advanced imaging findings to enable earlier, more accurate diagnosis and risk stratification, ultimately improving the dire prognosis associated with this condition.

## Preventive and therapeutic strategies and drugs for intestinal I/R injury

Advances in understanding the pathophysiology of intestinal I/R injury have catalyzed the shift from supportive care to mechanism-driven therapies. Current strategies aim to intercept the vicious cycle of oxidative stress, inflammatory cascades, and barrier failure. This section synthesizes these approaches into a coherent framework, spanning molecular interventions, regenerative medicine, microbiome modulation, and integrated clinical management.

## Targeting core injury mechanisms

### Antioxidant and Nrf2-activating strategies

Given that oxidative stress is a central executor of I/R damage, reinforcing endogenous antioxidant defenses has been a major therapeutic focus. The transcription factor Nrf2, as the master regulator of this response, represents a pivotal target. For instance, compounds such as isoquercitrin, dimethyl fumarate and bryostatin-1 confer protection by specifically activating the Nrf2/HO-1 pathway, thereby attenuating oxidative stress and secondary inflammation^136^, ^137^. Moreover, recent studies have revealed alternative mechanisms for mitigating oxidative damage. Coenzyme Q10 has been shown to alleviate intestinal I/R injury by inhibiting ferroptosis and lipid peroxidation^138^, while obacunone suppresses mitochondrial ROS and MIF signaling to inhibit RIPK1/RIPK3/MLKL-mediated necroptosis in intestinal I/R injury^139^. Similarly, the mitochondria-targeted antioxidant MitoQ mitigates intestinal epithelial cell apoptosis, an effect closely associated with the activation of Nrf2 signaling and the stabilization of mitochondrial integrity^15^. Beyond direct Nrf2 activators, other agents operate through complementary pathways: Glutamine alleviates injury by inhibiting the xanthine oxidase/uric acid axis^140^, while compounds like agmatine and naringin exert their effects by enhancing cellular glutathione reserves and reducing lipid peroxidation^141^, ^142^. Collectively, these findings underscore that boosting antioxidant capacity, particularly via the Nrf2 hub, is a validated strategy to mitigate the initial oxidative insult of I/R.

### Modulation of non-coding RNAs

The regulatory landscape of I/R injury is profoundly influenced by non-coding RNAs (ncRNAs), especially microRNAs (miRNAs) and circular RNAs (circRNAs), which fine-tune gene expression in processes like apoptosis and inflammation. Dysregulation of specific ncRNAs is therefore mechanistically linked to injury progression. For example, the downregulation of miR-378 post-I/R permits enhanced caspase-3 activity and apoptosis, whereas its overexpression provides protection^143^. Likewise, the overexpression of miR-339-5p diminishes ROS production by targeting p66Shc, thereby mitigating I/R-induced damage^144^. In addition to modulating cell death and oxidative stress, miR-146a and miR-381-3p are crucial for dampening the immunoinflammatory response and promoting epithelial repair^145^, ^146^. Furthermore, the circular RNA CircEZH2_005, whose expression decreases after injury, has been shown to promote the proliferation of Lgr5+ stem cells, facilitating mucosal regeneration^147^. Thus, these ncRNAs not only serve as promising diagnostic biomarkers but also represent a novel class of therapeutic targets for RNA-based interventions.

### Ischemic conditioning

IPO and IPC harness endogenous protective mechanisms, primarily by blunting the oxidative and inflammatory burst during early reperfusion. IPO exerts its benefit by reducing ROS generation and inhibiting the JAK/STAT signaling pathway, which in turn limits epithelial apoptosis^148^, ^149^. Complementarily, IPC appears to function through upregulation of aldose reductase and suppression of the TLR4-TRAF6 pathway^150^, ^151^. Critically, the protective window for these interventions is narrow; for instance, the synergy between IPO and IPC is most effective when applied during the initial moments of reperfusion^148^. This underscores the importance of timing in translating these powerful strategies into clinical practice.

## Advanced and regenerative therapies

### Stem cell and organoid-based therapy

Stem cell therapy, which involves the use of stem cells or their derivatives to repair damaged cellular structures, has gained widespread application in the treatment of various diseases, ranging from autoimmune disorders to organ ischemic injuries^152^. As a key component, mesenchymal stem cells (MSCs) have shown significant promise in addressing intestinal I/R injury, leveraging their self-renewal, multi-lineage differentiation, and potent immunomodulatory and paracrine functions^153^-^156^. It is noteworthy that although MSCs from different tissue sources (bone marrow, adipose tissue, and umbilical cord) share common core mechanisms like homing, immunomodulation, and paracrine signaling, their biological behaviors and therapeutic efficacy exhibit significant heterogeneity. This variability is primarily attributed to the distinct "tissue microenvironment imprint" carried by MSCs from each origin. For instance, bone marrow-derived MSCs can deliver miRNAs via extracellular vesicles to regulate the SREBF2/HMGB1 axis, thereby inhibiting ferroptosis^157^. Adipose-derived MSCs primarily exert their effects via the COX-2-PGE2 signaling axis^158^, and by regulating macrophage polarization^159^; furthermore, combining them with melatonin results in a synergistic therapeutic effect^160^. Umbilical cord-derived MSCs demonstrate notable anti-apoptotic and reparative capacities^161^. Furthermore, hair follicle-derived MSCs alleviate intestinal I/R injury by modulating oxidative stress responses and reducing apoptosis^162^. Collectively, these findings illustrate the tissue-specific mechanisms of action of MSCs from various sources, providing a theoretical basis for selecting optimal cell origins in clinical applications. Advancements in organoid technology further complement stem cell-based approaches. For example, Zhang *et al*. demonstrated that intestinal organoid transplantation in mice enhances survival, promotes the self-renewal of intestinal stem cells, and improves the immune microenvironment following I/R injury. This effect is associated with the secretion of L-Malic acid (MA), which polarizes anti-inflammatory M2 macrophages and restores interleukin-10 levels through the action of SOCS2^36^.

### Microbiota-targeted interventions

The gastrointestinal microbiota and their metabolites have demonstrated significant therapeutic potential in the treatment of AMI. Probiotic-based strategies, such as the administration of *Bifidobacterium bifidum* PRL2010, *Lactobacillus plantarum*, and *L. murinus*, have been shown to mitigate intestinal I/R injury by preventing bacterial translocation and preserving barrier function^30^, ^163^, ^164^. Similarly, the probiotic VSL#3 has been shown to effectively reduce local tissue damage caused by elevated inflammatory mediators and the recruitment of immune cells^165^. Furthermore, *Lactobacillus* amplifies DHAMaR1 conversion to attenuate intestinal I/R injury via decreasing pyroptosis^166^. Beyond probiotics, microbiota-induced natural antibodies play a crucial role in the host's inflammatory response to both sterile and infectious damage. The gut symbiotic microbiota enhances intestinal immune vigilance by modulating the formation and recruitment of NETs^167^.

Microbial metabolites also contribute significantly to intestinal protection. Short-chain fatty acids (SCFAs; e.g., butyrate, propionate, and acetate) help maintain mucosal integrity and attenuate neutrophil infiltration during I/R, highlighting their potential as preventive agents^168^, ^169^. The intestinal microbial metabolite pravastatin (PA) alleviates injury by enhancing interleukin-13 (IL-13) from innate lymphoid cells type 2 (ILC2)^41^, while milnacipran promotes intestinal tolerance via the AHR/ILC3/IL-22 pathway^170^. Propionate mitigates damage by modulating mast cell function through the AhR/Notch1 axis^171^, and microbiota-derived glutathione suppresses ferroptosis^172^. Additional metabolites such as capsiate (which activates TRPV1 to upregulate Gpx4 and inhibit ferroptosis)^21^, petroselinic acid (which activates the AMPK-mTOR pathway to suppress apoptosis)^173^, and Indole-3-lactic acid (which enhances epithelial stem cell function through YAP and Nrf2 activation and protects the gut-vascular barrier through AhR/Nrf2/STAT3-mediated claudin-2 downregulation)^174^, ^175^. These findings underscore the potential of the gut microbiome and its metabolic products in maintaining intestinal integrity and regulating immune responses, highlighting their significant impact on AMI treatment and suggesting new directions for future therapeutic strategies.

### Clinical management principles

The effective management of acute mesenteric ischemia (AMI), the stark clinical manifestation of I/R, demands a rapid, sequential, and multidisciplinary approach. Management must begin with aggressive fluid resuscitation to restore perfusion, accompanied by correction of acidosis and electrolyte imbalances. Crucially, early empirical broad-spectrum antibiotics are mandatory to mitigate the imminent risk of bacterial translocation and sepsis^176^-^178^. Therefore, a comprehensive approach that includes fluid management, correction of electrolyte and acid-base imbalances, hemodynamic monitoring, and proactive antibiotic treatment serves as the foundation for minimizing complications and optimizing the physiological state.

In the treatment of AMI, timely surgical intervention is critical; it aims to restore blood supply to the affected intestinal segment, resect all non-viable bowel areas, and preserve as much healthy intestine as possible. Surgical treatment is vital for ensuring intestinal viability, as failure to identify and manage non-viable intestine can lead to multi-organ dysfunction and increased patient mortality^179^. Acute peritonitis may indicate bowel infarction, necessitating immediate laparoscopy or laparotomy. Although surgical intervention enhances survival rates, mortality remains high in cases of acute arterial thrombosis. Nonetheless, nearly 40% of patients who undergo endovascular revascularization survive for one month post-operation, suggesting that endovascular treatment is an effective and cost-efficient management strategy for AMI^180^. Percutaneous stent placement and treatment of SMA occlusion yield significant benefits. Puncture aspiration and thrombolysis enhance outcomes in selected patient groups^181^. Subsequent laparoscopic examinations have minimal impact on outcomes^182^. While surgical treatment remains crucial, emerging endovascular strategies provide alternative options tailored to individual patient conditions. After successful intervention, long-term anticoagulation/antiplatelet therapy is required to prevent recurrence^183^, ^184^. Furthermore, lifelong monitoring with imaging surveillance is recommended to detect and manage late complications such as restenosis^180^.

The therapeutic arsenal for intestinal I/R injury is expanding from generic support to mechanism-targeted strategies, as synthesized in Table 1. From a translational perspective, several pathways and biomarkers emerging from experimental and early clinical studies show particular promise for near-term clinical application. Circulating markers of intestinal epithelial injury, including I-FABP, D-lactate, and citrulline, have been proposed as early indicators of intestinal barrier disruption. In parallel, inflammatory mediators such as HMGB1, pro-inflammatory cytokines, and markers of NET formation reflect systemic immune activation and correlate with distant organ injury. At the mechanistic level, signaling pathways involving TLR/MyD88 activation, oxidative stress responses, the Nrf2/HO-1 antioxidant pathway, and microbiota-derived metabolic signaling represent attractive therapeutic targets. Clinically feasible strategies to attenuate intestinal I/R-induced systemic injury may thus include interventions aimed at preserving microcirculatory integrity, modulating innate immune activation, or restoring gut microbiota homeostasis. Looking forward, the translational challenge lies in advancing ncRNA therapeutics and stem cell applications from bench to bedside, personalizing microbiota-targeted interventions, and, most importantly, integrating these novel adjuvant approaches into the time-sensitive clinical management framework to improve the prognosis of this condition.

## Others

Identifying early and specific biomarkers for gastrointestinal ischemic injury is essential^177^. Future research should concentrate on studying survivors of gastrointestinal ischemic injury to identify predictors of bowel resection and short bowel syndrome, as well as to develop visceral-mesenteric vasoactive agents for ischemia-related vasospasm. Currently, no single biomarker meets the gold standard diagnostic criteria of high sensitivity and specificity. Therefore, the comprehensive application of multiple laboratory indicators holds significant clinical value for assessing the likelihood of acute mesenteric ischemia. Future studies should continue to explore and validate novel biomarkers to improve diagnostic efficacy, and combining morphological techniques (such as microscopic imaging and tissue viability assessment) can further enhance diagnostic accuracy^185^. Establish 'intestinal stroke centers' that emphasize multidisciplinary collaboration, focusing on bowel resection, revascularization, and intensive care to prevent progression to multi-organ failure^179^. Despite the limited diagnostic capabilities of many current biomarkers, future research should aim to enhance diagnostic accuracy and efficiency through the integration of technologies such as artificial intelligence and machine learning. Strengthening the translation of basic research into clinical applications will facilitate the adoption of new therapies and technologies.

In conclusion, I/R injury represents a lethal, self-amplifying cascade in which microcirculatory failure and barrier disruption transform a localized ischemic event into a systemic inflammatory disorder. Acting as a central amplifier of immune and metabolic stress, the injured intestine promotes the dissemination of gut-derived danger signals, inflammatory mediators, and toxic metabolites, thereby driving distant organ injury and multiple organ dysfunction. This review systematically integrates the core mechanisms underlying intestinal I/R injury—including oxidative stress, regulated cell death, gut dysbiosis, and dysregulated immune signaling—within a unified pathophysiological framework linking local intestinal damage to systemic consequences. Based on these mechanistic insights, we summarize emerging diagnostic approaches and therapeutic strategies, such as ischemic conditioning, stem cell therapy, and microbiota-targeted interventions, that aim to interrupt key nodes of this injury cascade. Although timely revascularization remains the cornerstone of clinical management, precision interventions targeting specific components of the gut-centered inflammatory axis hold increasing translational promise. Future efforts should focus on translating these mechanistic advances into clinically validated tools through multi-omics-based risk stratification and personalized therapeutic strategies, with the ultimate goal of reducing the persistently high mortality associated with intestinal I/R injury.

## Figures and Tables

**Table 1:** Summary of Mechanism-Targeted Interventions and Strategies for Intestinal I/R Injury

Category	Intervention / Biomarker	Model & Timing / Dose	Mechanism / Key Effects	Reference
**Intestinal I/R Strategies**	Ischemic preconditioning (IPC)	SMA occlusion 5 min + reperfusion 5 min × 3	↓ ROS, ↑ SOD, ↓ inflammatory cytokines, ↓ apoptosis (caspase-3/Bax ↓, Bcl-2 ↑)	Wang, Zhidong et al.
	Ischemic postconditioning (IPoC)	Immediate 30 s reperfusion/30 s occlusion × 3	Similar to IPC; ineffective if delayed by 3 min	Liu, Ke-Xuan et al.
	Osthole	i.p. 10/50 mg/kg, 30 min before ischemia	Anti-inflammatory and antioxidant: ↓ ROS, ↑ SOD, ↓ neutrophil infiltration	Mo, Li-Qun et al.
	EGb 761 (Ginkgo extract)	p.o. 100 mg/kg for 7 days pre-modeling	Antioxidant, ↓ neutrophils and iNOS-NO	Liu, Ke-Xuan et al.
	Curcumin	i.v. 1/5 mg/kg, 50 min ischemia	NF-κB inhibition, ↓ IL-6, ICAM-1, neutrophil infiltration	Fan, Zhe et al.
	Fenofibrate	i.p. 100 mg/kg, 1 h before ischemia	PPARα agonist; inhibits NF-κB p65, reduces inflammation & apoptosis	Zhu, Qiankun et al.
	MSC-derived exosomes	i.v. 500 μL (3×10^6 MSCs) at reperfusion	TLR4/NF-κB suppression, ↓ inflammation	Liu, Jianpei et al.
**Distant Organ Protection**	Reba	p.o. 100 mg/kg/day	↑SIRT1, ↑β-catenin, ↑ FOXO1, ↓NF-κB p65	Elwany, Nisreen E et al.
	atenolol	intravenous atenolol infusion (2 mg/kg)	↑malonaldehyde (p=0.001), ↓ TNF-α (p=0.001)	Okada, Mieko et al.
	SB239063 (p38MAPK inhibitor)	i.p. 10 mg/kg, 1 h post-op	↓ p38MAPK → ↓ IL-1β / AQP4, ↓ lung injury	Zheng, De-Yi et al.
	FK866 (visfatin inhibitor)	i.p. 10 mg/kg at reperfusion	↓ Visfatin → NF-κB suppression, ↓ inflammation & apoptosis	Matsuda, Akihisa et al.
	Melatonin → Brain	i.p. 75/150 mg/kg at reperfusion	TLR4/MyD88 inhibition in microglia, ↓ neuroinflammation & apoptosis	Yang, Bo et al.
**Diagnostics / Imaging**	CT / CTA	MDCT / Biphasic MDCT Angiography	AMI diagnosis: Sensitivity 93-96%, Specificity 96-100%; non-enhancement predicts necrosis	Menke, Jan.
	D-dimer	Serum test	Sensitivity 96%, specificity 18-40%; > 1.5 mg/L suggests SMA occlusion risk	Chiu, Yu-Hui et al.
	I-FABP	Plasma / urine test	Plasma: Sens 79%, Spec 91%; Urine AUC 0.88; > 3.1 ng/mL for small intestine ischemia	Kanda, T et al.
	Myoglobin, AST, PCT, PALM score	Combined scoring system	AUROC 0.93; PALM ≥14 predicts high risk	Zogheib, Elie et al.
**Clinical Treatments**	Crystalloid resuscitation	Up to 100 mL/kg or 32 mL/kg/h	Stabilizes hemodynamics, corrects acidosis/hyperkalemia	Lagoa, Claudio Esteves et al.
	Oral antibiotics	Gentamicin 80 mg/d + Metronidazole 1.5 g/d	↓ ITIN risk (HR 0.16)	Nuzzo, Alexandre et al.
	Surgery / endovascular revascularization	Open vs PTA + stenting	Revascularization reduces 30-day mortality (42% vs 62%)	Erben, Young et al.
	Papaverine ± PGE1	Intra-arterial SMA infusion	↑ SMA perfusion; improved survival in NOMI (risk diff -11.6%)	Boley, S J et al.
	Teduglutide (GLP-2 analog)	s.c. 0.05 mg/kg/day	↓ TPN by 4.4 L/week; ↑ mucosal mass (citrulline)	Jeppesen, Palle B et al.
	IVIG	i.v. 0.5-1 g/kg	Clears C3b/C5a, inhibits complement deposition, protects local & distant organs	Anderson, Jimie et al.
	Probiotics / SCFA (e.g., B. bifidum, VSL#3, butyrate)	Multiple strains and metabolites	↓ BT, inflammation, apoptosis, NETs; ↑ barrier, IL-10/IgG; ↓ TLR4/TRIF	Qiao, Yingli et al.

## Data Availability

The authors confirm that the data supporting the findings of this study are available within the article.
